# Formation and Inhibition of N^ε^-(Carboxymethyl)lysine in Saccharide-Lysine Model Systems during Microwave Heating

**DOI:** 10.3390/molecules171112758

**Published:** 2012-10-31

**Authors:** Lin Li, Lipeng Han, Quanyi Fu, Yuting Li, Zhili Liang, Jianyu Su, Bing Li

**Affiliations:** 1 College of Light Industry and Food Sciences, South China University of Technology, Guangzhou 510640, China; 2 Guangdong Province Key Laboratory for Green Processing of Natural Products and Product Safety, Guangzhou 510640, China

**Keywords:** N^ε^-(carboxymethyl)lysine, microwave heating, formation, inhibition

## Abstract

N^ε^-(carboxymethyl) lysine (CML) is the most abundant advanced glycation end product (AGE), and frequently selected as an AGEs marker in laboratory studies. In this paper, the formation and inhibition of N^ε^-(carboxymethyl)lysine in saccharide-lysine model systems during microwave heating have been studied. The microwave heating treatment significantly promoted the formation of CML during Maillard reactions, which was related to the reaction temperature, time and type of saccharide. The order of CML formation for different saccharides was lactose > glucose > sucrose. Then, the inhibition effect on CML by five inhibitors was further examined. According to the results, ascorbic acid and tocopherol did not affect inhibition of CML, in contrast, thiamin, rutin and quercetin inhibited CML formation, and the inhibitory effects were concentration dependent.

## 1. Introduction

**Scheme 1:**
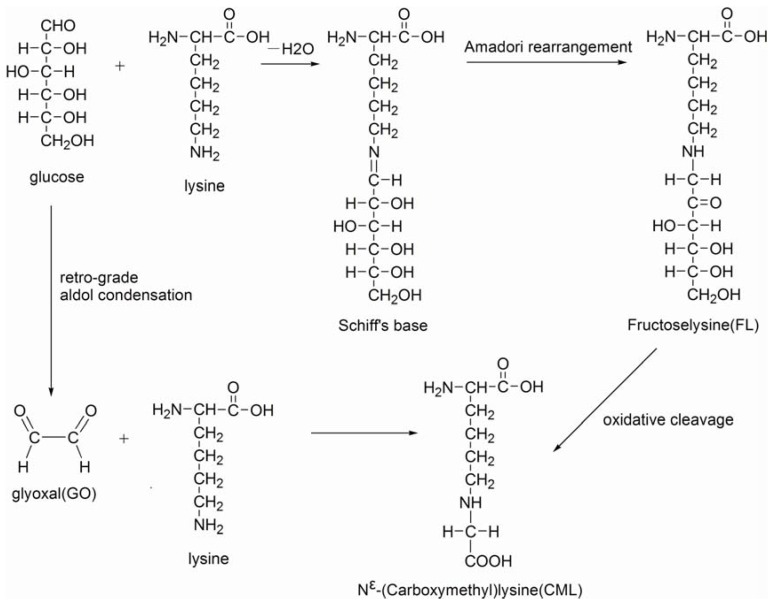
Possible pathway for the formation of CML in food systems.

Advanced glycation end products (AGEs) are a group of complex heterogeneous molecules, formed from the nonenzymatic reactions of sugars or their oxidation products with the free amino groups of proteins or the oxidation of lipids [1,2,3]. AGEs are regarded a major pathological component in diabetes-related complications [4,5,6]. Approximately 10% of diet-derived AGEs were confirmed to be absorbed into circulation and correlate with tissue AGEs levels by many studies [7,8]. N^ε^-(carboxymethyl)lysine (CML), N^ε^-(carboxyethyl)lysine (CEL), hydroimidazolones (HIs), pentosidine and pyrraline are the better characterized and most widely studied AGEs [9,10]. As the most abundant AGE, CML, which was also the first identified in dietary food, has been frequently selected as an AGE marker in laboratory studies [11,12,13]. Scheme 1 shows the possible pathway for the formation of CML in food systems. Due to the potential harm to humans, dietary CML has been defined as heat-induced processing glycotoxins.

As a fast and convenient heat processing method, microwave heating of food has been becoming increasingly common in food processing. Through molecular interactions, microwave energy transfer occurs via conversion of electromagnetic energy to thermal energy. Recently, microwaves have been shown to significantly promote the Maillard reaction and other chemical reactions [14]. Pagnotta found there was a significant difference in the mutarotation of α-D-glucose to β-D-glucose caused by microwave heating and conventional heating. The proportion of α-D-glucose increased with time under microwave heating, however, this phenomenon did not happen under conventional heating [15]. Furthermore, α-D-glucose was more unstable and prone to Maillard reactions than β-D-glucose.

The Maillard reaction in food systems has a direct impact on the formation of CML in dietary foods, but few studies have been concentrated on the CML formation and inhibition mechanisms during microwave heating. In this study, the microwave heating method of synthesis of CML in saccharide-lysine model systems had been studied, including the determination of the formation of CML in saccharide-lysine model systems at temperature and time relevant to microwave heating treatment, as well as the inhibition mechasnism of five substances (ascorbic acid, tocopherol, thiamin, rutin and quercetin) on CML.

## 2. Results and Discussion

### 2.1. Formation of CML by Conventional Heating

#### 2.1.1. Temperature of Heating

**Figure 1:**
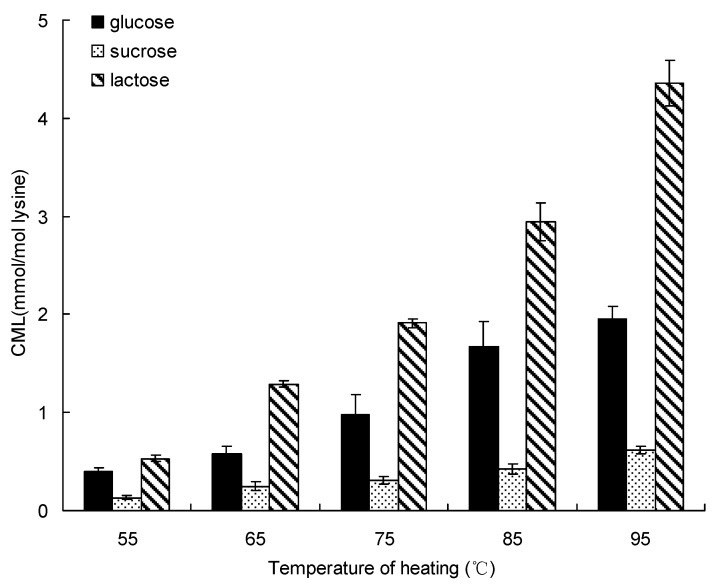
Formation of CML in saccharide-lysine by temperature. Data points are the mean of triplicate measurements ± SD.

Processing temperature is one of the most important factors during thermal treatment, Figure 1 shows the effect of water bath heating treatment on formation of CML in the saccharide (glucose, sucrose and lactose)-lysine model system below 100 °C. The same trends were observed in these model systems, all formations of CML incubated by different saccharides was increased from 55 °C to 95 °C for 20 min. The formation of CML in the glucose-lysine model systems gave the highest amount (1.96 ± 0.12 mmol/mol lysine) at 95 °C for 20 min. Sucrose produced the lowest amount of CML, which increased to a highest amount of 0.62 ± 0.04 mmol/mol lysine at 95 °C for 20 min. Lactose produced the highest amount of CML, ranging from 0.53 ± 0.03 mmol/mol lysine at 55 °C to the a highest amount of 4.36 ± 0.23 mmol/mol lysine at 95 °C for 20 min.

#### 2.1.2. Time of Heating

**Figure 2:**
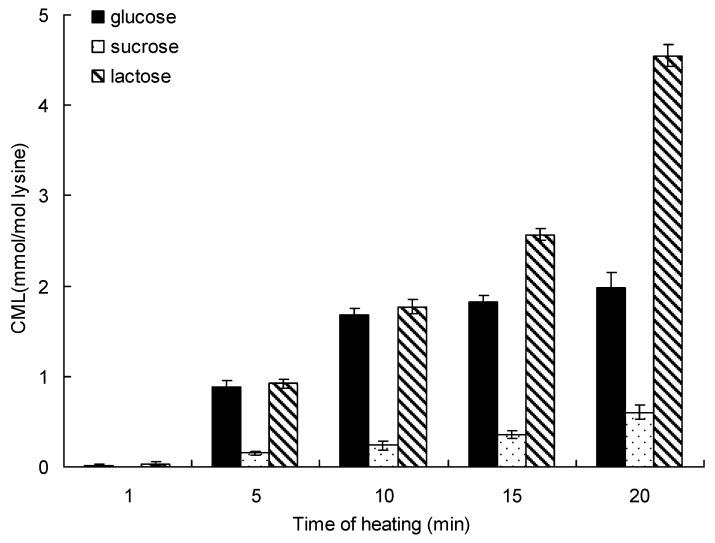
Formation of CML in saccharide-lysine over time. Data points are the mean of triplicate measurements ± SD.

Heating time is another important factor that affects the formation of CML. Figure 2 shows the formation of CML in the saccharide-lysine model systems incubated in a water bath for up to 20 min at 95 °C. In the lactose-lysine model system, the CML content increased significantly from 0.03 ± 0.02 mmol/mol lysine for 1 min to 4.36 ± 0.23 mmol/mol lysine after 20 min at 95 °C. The glucose-lysine and sucrose-lysine model systems show the same trends, whereby the formation of CML increased with heating time. The formation of CML incubated by lactose was 2.23-time and 7.03-times higher than glucose and sucrose for 20 min at 95 °C, respectively. Like in the effect of temperature, the order of reactivity for the formation of CML was lactose > glucose > sucrose.

Reducing sugars (*i.e.*, glucose, galactose and lactose) are more easily thermally degraded by dienol structure interchange than other saccharides in the Maillard reaction [16]. The reducing configuration and open chair form of the saccharide would be a prerequisite for CML formation. However, during the heat treatment, sucrose and lactose were hydrolyzed into glucose and fructose, glucose and galactose, respectively. Due to the reducing sugar units, sucrose and lactose formed CML during the Maillard reaction. As was reported by Courel, glucose produced significantly higher amounts of CML *versus* sucrose up to 230 °C; about 2.5 times more CML was formed at 230 °C [17]. Recent studies showed that sucrose hydrolysis seems to be the limiting step of the saccharide degradation process at low temperatures [18]. Furthermore, as a non-reducing sugar, sucrose cannot be oxidized to GO or form a Schiff base with lysine, thus, sucrose has difficulty in forming CML at normal temperatures.

The difference in the amount of CML formed from sucrose and lactose may be because sucrose is more stable than lactose when heated, and lactose is a reducing disaccharide. The presence of the reducing disaccharide lactose is favorable to glycation reactions. Lactose will be directly oxidized to GO or fructoselysine (FL), and some of these intermediates ultimately become CML. On the other hand, lactose consists of reducing monosaccharide glucose and galactose units. With the reactive carbonyl group on the glucose and galactose unit, in the Maillard reaction lactose can react with available amino groups of the protein to form an N-substituted glycosyl amine. Recent research found the average amount of CML in lactose hydrolysed infant formulas were approximately 40–80% higher than in lactose-free hydrolysed infant formulas [19]. This may infer that reducing sugars form more CML than non-reducing sugars.

### 2.2. Formation of CML by Microwave Heating

#### 2.2.1. Temperature of Heating

**Figure 3:**
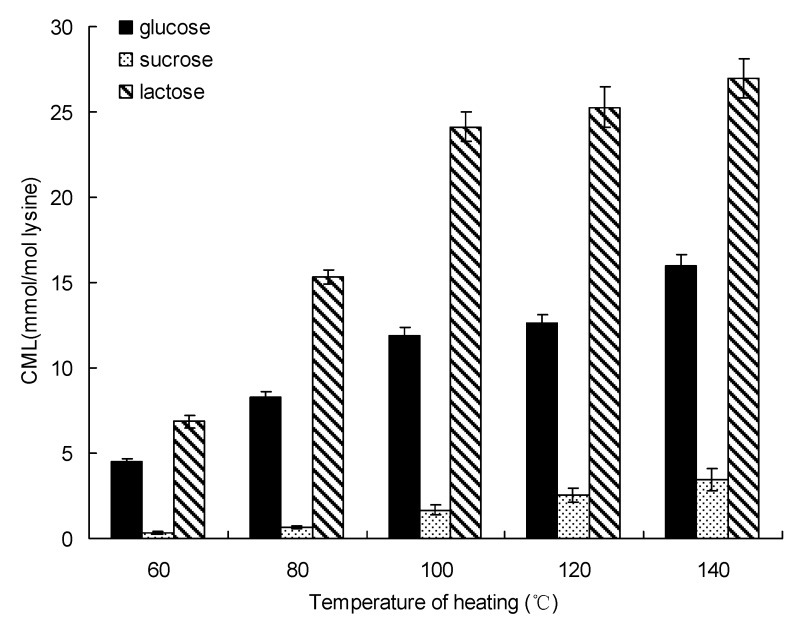
Formation of CML in saccharide-lysine by temperature. Data points are the mean of triplicate measurements ± SD.

The formation of CML changed significantly with heating temperature (Figure 3). The same trends also existed in glucose-lysine, sucrose-lysine and lactose-lysine model systems. After 20 min of heating, the formation of CML in glucose-lysine model system increased from 4.49 ± 0.21 mmol/mol lysine at 60 °C to 16.01 ± 0.61 mmol/mol lysine at 140 °C, while the concentration of CML in the sucrose-lysine model system was only 3.45 ± 0.67 mmol/mol lysine after 20 min of heating at 140 °C. The amount of CML increased from 6.85 ± 0.35 mmol/mol lysine for lactose at 60 °C to 26.99 ± 1.13 mmol/mol lysine at 140 °C.

#### 2.2.2. Time of Heating

**Figure 4:**
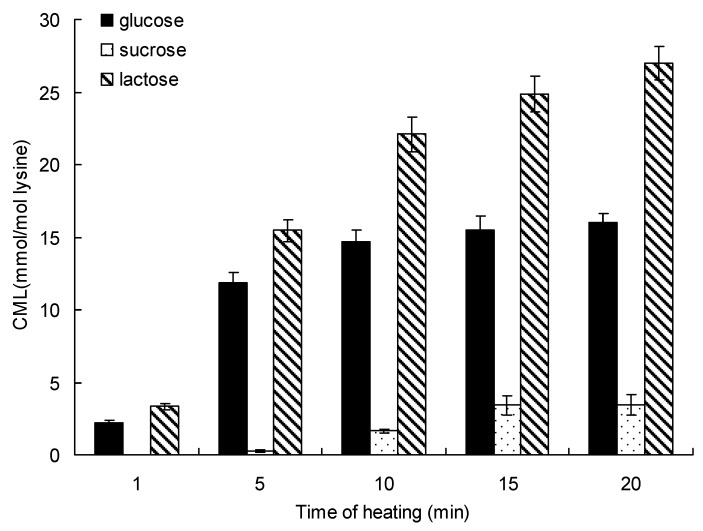
Formation of CML in saccharide-lysine over time. Data points are the mean of triplicate measurements ± SD.

Figure 4 shows the relationship between production and heating time on the formation of CML for up to 20 min at 140 °C. The initial formation of CML (determined over the initial 1 min) was 2.25 ± 0.12 mmol/mol lysine in the glucose-lysine model systems and 3.30 ± 0.24 mmol/mol lysine in the lactose-lysine model systems, and no CML was detected in the sucrose-lysine model systems. At 20 min of microwave heating, the order of CML formation was lactose > glucose > sucrose. Lactose and lysine produced the highest amount of CML, and the maximum formation of CML incubated in lactose-lysine model systems was 1.69-fold and 7.89-fold higher than in the glucose-lysine and sucrose-lysine model systems.

Microwave heating is a dielectric heating, which is related to the molecular motion via migration of ions and rotation of dipoles. Thus, microwaves do not directly disrupt the chemical bonds of molecular structures. As observed in previous studies, compared with conventional heating, microwave heating affected the mutarotation of α-D-glucose, which was more unstable and prone to Maillard reactions than β-D-glucose [15]. The Maillard reaction in food system directly affected the formation of CML. In this paper, the initial period of microwave heating system, the system temperature increased rapidly, the free radical density was obviously increased, the chain transfer responds by speeding up, and large amounts of saccharides were oxidized to glyoxal (GO) or form a Schiff base with lysine, and some of these intermediates ultimately become CML in a short time [20,21,22].

### 2.3. Inhibition of CML in Glucose-Lysine Model Systems during Microwave Heating

#### 2.3.1. Effect of Inhibitors on CML Formation

**Figure 5:**
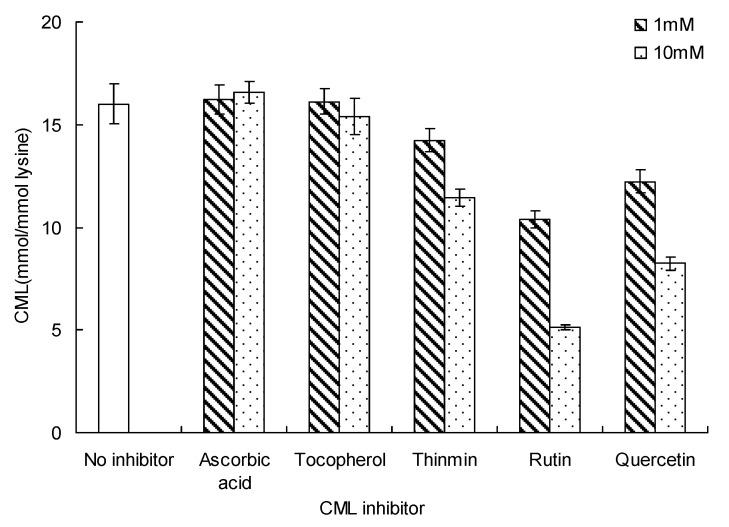
Inhibition of CML by vitamins and flavonoids in glucose-lysine model systems during microwave heating, at 140 °C for 20 min. Data points are the mean of triplicate measurements ± SD.

The effect of different inhibitors on CML formation is shown in Figure 5. Among the three vitamins tested, ascorbic acid and tocopherol did not affect CML formation in the glucose-lysine model system during microwave heating, at 140 °C for 20 min. In contrast, only one vitamin (thiamin) and all of the flavonoids tested (rutin and quercetin) showed a good inhibitory effect on CML formation. CML concentration in the absence of any inhibitor was 16.01 ± 0.01 mmol/mol lysine. CML concentrations with thiamin were 14.24 ± 0.56 and 11.45 ± 0.42 mmol/mol lysine (concentration of thiamin = 1 and 10 mM, respectively).The lowest concentrations of CML were appeared in the presence of 10 mM rutin (5.41 ± 0.15 mmol/mol lysine). Ten mM quercetin gave similar concentrations of CML (8.25 ± 0.31 mmol/mol lysine), about 52.45% lower than in the presence of rutin. The inhibitory effect of flavonoids on CML formation was higher than that of vitamins.

In this paper, the three vitamins exhibited great differences in inhibition of CML. Ascorbic acid and tocopherol did not affect inhibition of CML, despite the high concentration of ascorbic acid and tocopherol used. In contrast, thiamin, rutin and quercetin inhibited CML formation, and the inhibitory effect was concentration dependent.

#### 2.3.2. Effect of Inhibition Temperature and Time on Inhibition of CML

**Figure 6:**
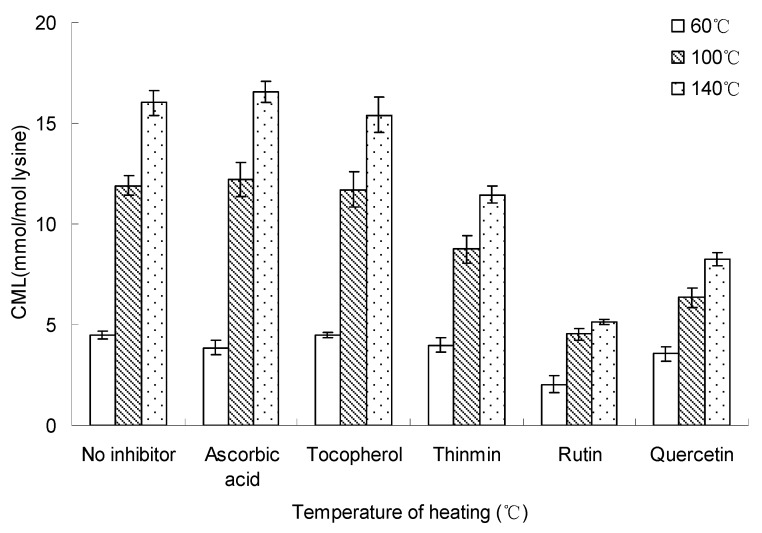
Inhibition temperature on inhibiting the formation of CML in glucose-lysine model systems during microwave heating. The heating time was 20 min and the concentration of inhibitor was 10 mM. Data points are the mean of triplicate measurements ± SD.

**Figure 7:**
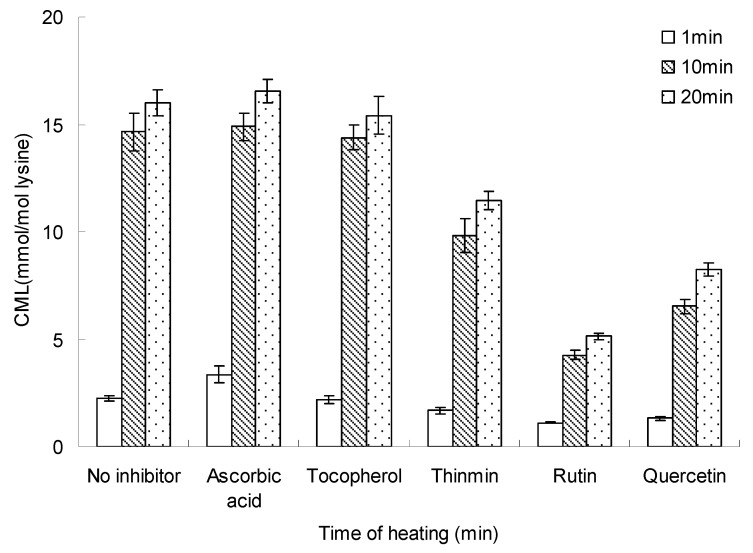
Inhibition time on inhibiting the formation of CML in glucose-lysine model systems during microwave heating. The heating time was 140 °C and the concentration of inhibition was 10 mM. Data points are the mean of triplicate measurements ± SD.

Figure 6 and Figure 7 show the heating temperature and time effect on inhibition of the formation of CML in glucose-lysine model systems (concentration of inhibitor = 10 mM). The figures showed the formation of CML increased with the increase of heating temperature and time, however, rutin and quercetin significantly reduced the formation of CML relative to blank control with no inhibitor, while ascorbic acid and tocopherol showed no effect on inhibition of CML, and ascorbic acid made a small contribution to the formation of CML.

Ascorbic acid and tocopherol are common antioxidants and free radical scavengers, capable of reacting directly with various free radicals created by the hydrogen atoms in structures. Thus, they would theoretically have the capacity to inhibit CML formation. However, in this paper, ascorbic acid and tocopherol showed less effect on CML inhibition. Although ascorbic acid is a widely used antioxidant, that provides free radical scavenging activity towards free radicals, it did not inhibit CML formation in a glucose-lysine model system used in the paper. Due to the carbonyl group of ascorbic acid, it is CML precursor. Ascorbic acid may be oxidized to yield erythrose, which may form a Schiff base or Amadori rearrangement product with lysine, and undergo oxidative cleavage to CML [23]. Tocopherol is a lipid-soluble phenolic antioxidant, and had been previously proven to not inhibit CML formation in the glycated model system, which may be due to the insolubility of tocopherol in the phosphate buffer system used [24].

Thiamine is a competitor in the formation of CML in glucose-lysine model systems, and inhibits CML formation depending on concentration. Thiamine consists of substituted pyrimidine and thiazole rings, and the amino group of thiamine can compete with the amino group of lysine residues, and react with the carbonyl group of a reducing sugar during the Maillard reaction and protein glycation [25]. Furthermore, the CML inhibition effect of thiamine is possibly through the reaction with the intermediates (GO and MGO) of CML [26]. Due to a competitive reaction with the ε-amino group of lysine residues or intermediate of CML, thiamine has a strong anti-glycation effect *in vitro*.

**Figure 8:**
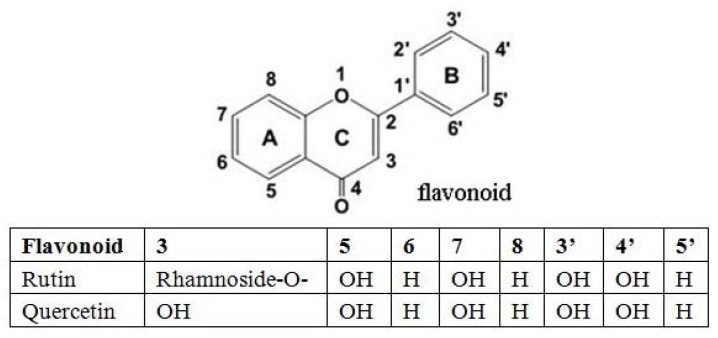
The structures of rutin and quercetin.

The structures of rutin and quercetin are given in Figure 8. Rutin and quercetin containing vicinyl dihydroxyl groups in the B-ring were effective inhibitors in the autoxidation of glucose, and inhibited the formation of AGEs such as CML, pentosidine in a cross-linked fluorescence *in vitro* model, and their inhibitory effect on CML formation was concentration dependent [27]. As powerful free radical scavengers and anti-glycation agents, rutin and quercetin inhibited CML formation at all of different stages of glycation, including autoxidation of glucose, retro-aldol condensation of glucose and the Schiff base, the oxidative degradation of Amadori products, GO formation and oxidative degradation of Amadori products to CML [28,29]. Furthermore, rutin and quercetin could scavenge reactive oxygen species (superoxide and H_2_O_2_) and reactive carbonyl groups, which mainly inhibit the CML intermediate (GO) formation [30].

## 3. Experimental

### 3.1. Preparation of Saccharide-Lysine Conventional Heating Model Systems

The model systems were incubated in a water bath (Jintan, China). The saccharide and lysine solutions dissolved in phosphate buffer (0.2M; pH 6.8) were added into glass vials. All glass vials were carefully sealed. During incubation, glass vials were left in the shaking water bath for the appropriate time. The fluctuation range of set internal temperature was less than ±1 °C.

### 3.2. Preparation of Saccharide-Lysine Microwave Heating Model Systems

These saccharide/lysine model systems were incubated by a microwave digestion labstation (Ethos1, Italy). In the microwave digestion labstation system, the reaction temperature and time can be controlled with a digital intelligent control panel. A high sensitivity IR sensor was used for monitoring the surface and internal temperature of all microwave digestion vessels. The fluctuation range of set internal temperature and internal vapor pressure was less than ±1 °C and ±1 bar. In the saccharide/lysine microwave heating model systems, the saccharide/lysine solutions dissolved in phosphate buffer (0.2M; pH 6.8) were aliquoted into microwave digestion vessels, which were carefully sealed. After a given heating time, all samples were cooled in ice water to stop any further reaction and stored at −80 °C, prior to analysis.

Model 1: *Effect of Temperature*. In the first model system, solutions of saccharide (10 mM) and lysine (10 mM) in phosphate buffer (NaH_2_PO_4_ and Na_2_HPO_4_, 0.2M, pH 6.8) were heated for 20 min at 60, 80, 100, 120 and 140 °C. The temperature programming of the microwave digestion labstation at different set heating temperature in this paper is shown in Table 1.

Model 2: *Effect of Time.* Solutions of saccharide (10 mM) and lysine (10 mM) in phosphate buffer (NaH_2_PO_4_ and Na_2_HPO_4_, 0.2M, pH 6.8) were heated at 140 °C for 1, 5, 10, 15 and 20 min. The time programming of the microwave digestion labstation at different set heating time in this paper is shown in Table 2.

Glucose, sucrose and lactose were the various saccharides tested. The glucose-lysine, sucrose-lysine or lactose-lysine model systems in sealed digestion vessels were heated by the microwave digestion labstation using the programmed temperature and time settings. All tests were performed in triplicate.

### 3.3. Inhibition of CML by Vitamin and Flavonoid

Glucose (10 mM), lysine (10 mM) and inhibitors (1 mM or 10 mM) in phosphate buffer (0.2M, pH 6.8) were heated by microwave heating at 140 °C for 20 min. The set temperature reached 140 °C for 5 min. The inhibitors included three vitamins (thiamin, ascorbic acid and tocopherol) and two flavonoids (rutin and quercetin). We also tested the effect of inhibition temperature and time on inhibiting the formation of CML with 10 mM inhibitor. All samples were cooled in ice water to stop any further reaction and stored at −80 °C, prior to analysis. The purification step was described in Section 3.4.

### 3.4. Purification by Solid Phase Extraction

A 100 μL volume of incubated solution was reduced overnight at 4 °C by sodium borohydride solution (0.2M, 100 μL). Each sample was centrifuged at 20,000 rpm for 60 min in an ultracentrifuge at −4 °C (Sigma, Germany), the isolated solution was eluted on a 6 mL Cleanert C18 cartridge (Agela, China). The Cleanert C18 was washed with 3 mL of methanol-water–formic acid (10:90:0.1, v/v/v). The eluate was dried under vacuum and reconstituted in 0.1% aqueous formic acid (1 mL) at −4 °C prior to analysis.

### 3.5. Determination of CML Content by HPLC-MS/MS

The HPLC-MS/MS analysis for the determination of CML content was as described by Ahmed and Assar with some modifications [31,32]. The chromatographic system consisted of a HPLC system (Waters, USA) coupled to a Waters multi-moe ionization mass spectrometer using the electrospray positive ionization (ESI+) method. Separations were conducted on a reversed phase Atlantis T_3_ C_18_ analytical column (150 mm × 4.6 mm, 5 μm particle size; Waters), conditioned at 25 °C. A 10 μL volume of eluate or CML was injected into the reversed column, and eluted with a mixture of methanol-water-formic acid (10:90:0.1, v/v/v) at a flow rate of 0.5 mL/min. Sample peaks corresponding to CML were calculated by using the equation of the relevant standard curves, the correlation coefficient (R^2^) value was ≥0.995 for all calibration curves.

## 4. Conclusions

Microwave heating significantly promoted the formation of CML during Maillard reactions, which was related to the reaction temperature, time and kind of saccharide used. In conclusion, the microwave heating treatment affected the performance of formation of CML in a short time, with reducing sugars (glucose and lactose) being more easily thermally degraded by dienol structure interchange than other saccharides in the Maillard reactions, and more CML being formed than with a non-reducing sugar (sucrose). 

In this paper, the inhibitory effects of five inhibitors on CML formation have been investigated. Three of them (thiamin, rutin and quercetin) exhibited inhibition effects on CML formation, and the inhibitory effect was concentration dependent. Thiamin was a competitor in the formation of CML in glucose-lysine model systems, and inhibited CML formation dependent on concentration. Rutin and quercetin containing vicinyl dihydroxyl groups in the B-ring were effective inhibitors of CML formation at all of the different stages of glycation.

## Figures and Tables

**Table 1:** Temperature programming of different set heating temperature.

Heating temperature (°C)	Temperature programming
60	RT→60 °C (5 min), 60 °C (20 min)
80	RT→80 °C (5 min), 80 °C (20 min)
100	RT→100 °C (5 min), 100 °C (20 min)
120	RT→120 °C (5 min), 120 °C (20 min)
140	RT→140 °C (5 min), 140 °C (20 min)

RT, room temperature.

**Table 2:** Time programming of different set heating time.

Heating time (min)	Time programming
1	RT→140 °C (5 min), 140 °C (1 min)
5	RT→140 °C (5 min), 140 °C (5 min)
10	RT→140 °C (5 min), 140 °C (10 min)
15	RT→140 °C (5 min), 140 °C (15 min)
20	RT→140 °C (5 min), 140 °C (20 min)

RT, room temperature.
